# Complex Primary Total Knee Arthroplasty in a Patient with Achondroplasia, Osteoarthritis, and Severe Coronal Instability

**DOI:** 10.1016/j.artd.2020.12.023

**Published:** 2021-02-24

**Authors:** Ryan Stancil, Michael Goldberg, Maryse Bouchard, Adam Sassoon

**Affiliations:** a Department of Orthopaedics and Sports Medicine, University of Washington, Seattle, WA, USA; b Department of Orthopaedic Surgery, Seattle Children’s Hospital, Seattle, WA, USA; c Division of Orthopedics, Hospital for Sick Children, Toronto, Ontario, Canada; d Department of Orthopaedic Surgery, University of California, Los Angeles, Los Angeles, CA, USA

**Keywords:** Achondroplasia, Complex primary total knee arthroplasty, Genu varum, Ligamentous laxity

## Abstract

Genu varum in patients with achondroplasia is common and is often exacerbated by the associated generalized ligamentous laxity. Despite this, development of knee osteoarthritis is rare. There are only a few previously published case reports of total knee arthroplasty in this population. We present 2-year follow-up of a patient with achondroplasia who underwent staged bilateral primary total knee arthroplasties using hinged components. Technical considerations and careful preoperative planning are required in patients with achondroplasia given their small skeletal stature, metaphyseal deformities, and ligamentous laxity.

## Introduction

Achondroplasia is the most common skeletal dysplasia. It is an autosomal dominant form of short-limbed dwarfism caused by a mutation in the fibroblast growth factor receptor 3 gene on chromosome 4p16.3. [1]. The mutation is spontaneous in 90% of cases and suppresses chondrocyte growth and proliferation. The classic phenotype of achondroplasia is characterized by a long-trunk short-limb short stature. Limb shortening is rhizomelic, predominantly affecting the proximal long bones such as the humerus and femur. Other typical features include macrocephaly with frontal bossing, midface hypoplasia, trident hands, and generalized ligamentous laxity of most joints. The knee is most commonly affected [2].

The fibroblast growth factor receptor-3 mutation in achondroplasia causes abnormal enchondral ossification affecting the physes in long bones and growth of flat bones such as the base of the skull and the posterior elements of the spine, thereby accounting for the classic features of short limbs, craniofacial differences, and spinal stenosis. Intramembranous ossification is unaffected, therefore normal bone width is preserved [3]. Most importantly, this mutation does not adversely impact articular cartilage, as it is common in other skeletal dysplasias such as type 2 collagenopathies (ie, spondyloepiphyseal dysplasia). This is likely why end-stage osteoarthritis is rare in patients with achondroplasia [3].

In typical, patients with achondroplasia develop a varus lower limb deformity. Bony dysplasia causing proximal and distal femoral varus, lateral tibial bowing, and fibular overgrowth contribute to the overall varus mechanical alignment. The soft tissue laxity of the knee joint can further exacerbate the varus deformity [4,5]. Patellar dislocation has been reported in a series of patients because of a combination of soft tissue laxity, abnormal gait kinematics, and a shallow trochlea [6,7]. A decreased tibial sagittal slope compared to population controls can occur causing concurrent genu recurvatum [8]. Some patients with achondroplasia may elect to undergo realignment procedures via high tibial osteotomies or less often distal femoral osteotomies in addition to limb-lengthening surgeries [9]. This is important to note because these procedures can alter the anatomic axes of the femur and tibia. In typical, femoral lengthening via an external fixator will lead to varus, whereas lengthening via an intramedullary device will lead to valgus. After an osteotomy, tibial deformity can occur in either varus or valgus if a plate construct is used. With an intramedullary device, however, valgus and procurvatum is the most common deformity.

Despite the severity and frequency of genu varum deformity in achondroplasia, reports of end-stage osteoarthritis and subsequent total knee arthroplasty (TKA) are rare. One case report describes staged bilateral TKAs with ipsilateral simultaneous closing wedge metaphyseal femoral osteotomies in a patient with achondroplasia. Both TKA implants used were rotating-hinge implants with long stems [10]. Kim et al. published a series of TKAs performed in patients with skeletal dysplasias, including 5 TKAs performed in 3 patients with achondroplasia [11]. All these patients had varus deformities requiring medial soft tissue releases. In some cases, the authors used computer navigation techniques given the patients’ bony deformity and used either mobile bearing or posterior stabilized implants [11].

We report a 45-year-old female with achondroplasia and the end-stage knee osteoarthritis secondary to a severe coronal plane instability. She required staged bilateral TKAs with hinged components using all-polyethylene tibial components given her severe soft ligamentous laxity and instability. We additionally present our surgical technique, as it required unconventional modifications. Her small skeletal stature precluded standard instrumentation, necessitating “free-hand” preparation of the femur and intramedullary tibial resection.

## Case history

A 45-year-old female patient with achondroplasia presented to an outpatient arthroplasty clinic with a 3-year history of bilateral knee pain and progressive varus deformities. She was previously active and participated in sports such as cross-country skiing. With the progressive worsening of her knee osteoarthritis, she became limited to using a walker for ambulation. She struggled to continue her work as an attorney and independently performing her activities for daily living despite anti-inflammatories, physical therapy, and corticosteroid injections. Her only concurrent medical issue was depression, for which she took a selective serotonin reuptake inhibitor. Her past surgical history included bilateral tibial osteotomies with subsequent removal of hardware as a child to address a limb deformity. She could not recall details of the procedure, and no historical operative records were available.

On examination, she was 1.14 m tall and weighed 49 kg, with a BMI of 34.5. Her gait examination was notable for a varus thrust bilaterally, right worse than left. Her knee range of motion was 0° to 100° on the left and −2 to 95° on the right, demonstrating mild recurvatum. She had approximately 20° of bilateral resting varus alignment, which was correctable to neutral with valgus stress. She had no lateral restraints with varus stress bilaterally.

**Figure 1:**
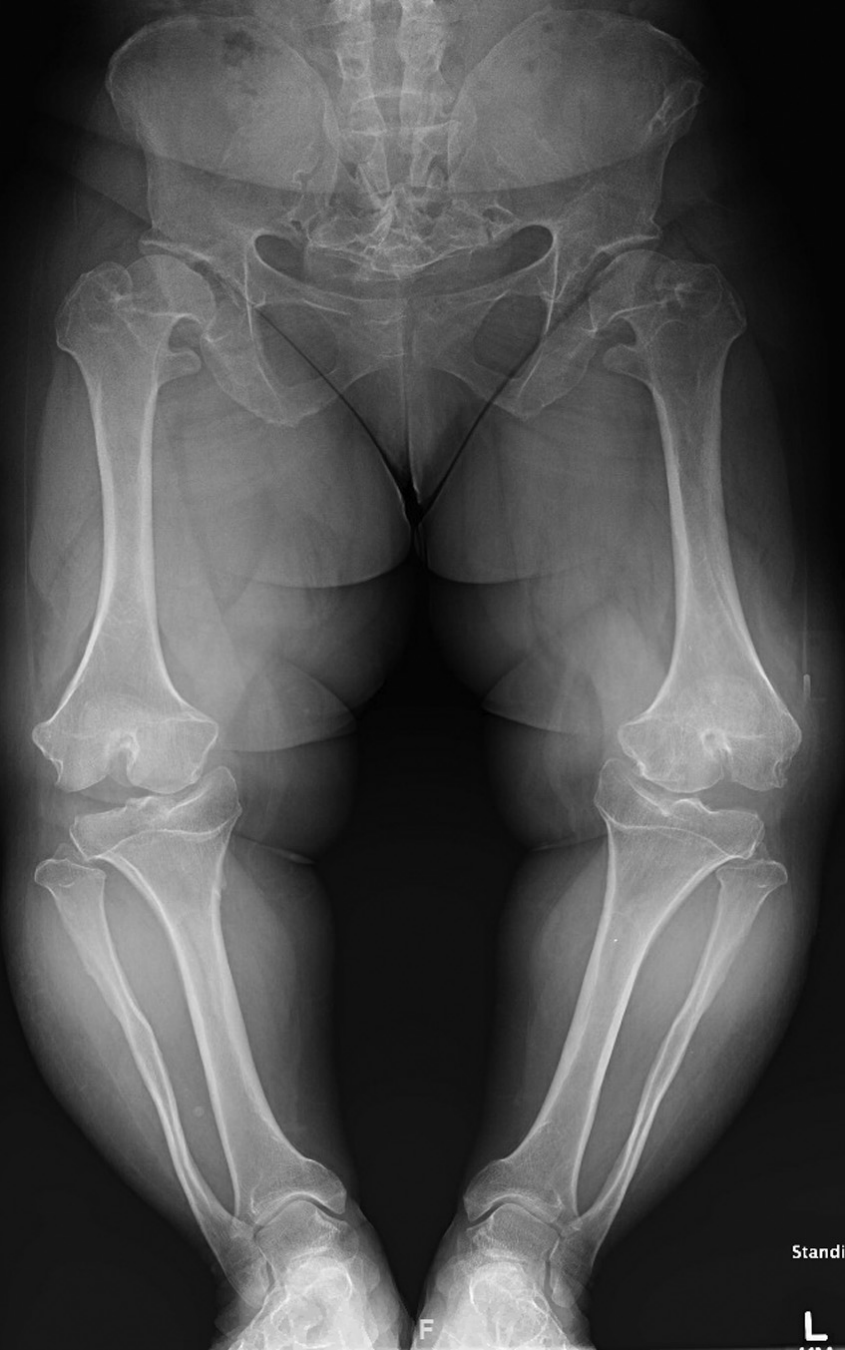
AP bone length radiograph. Characteristic rhizomelic shortening and metaphyseal changes associated with achondroplasia. Notable medial joint space narrowing with lateral widening suggestive of instability.

Radiographs, including standing alignment films, showed end-stage medial compartment osteoarthritis and varus limb alignment. Her mechanical axis passed medial to her knees bilaterally (Fig. 1). Given her worsening quality of life, her significant joint instability, and failure of nonsurgical modalities, staged bilateral TKA was indicated.

Numerous technical options for TKA were considered given the patient’s ligamentous laxity and abnormal bony morphology. Stemmed rotating hinge components were selected to address her instability and mitigate the subsequent risk of aseptic loosening. In addition, this system allowed for placement of pediatric sized all-polyethylene tibial implants, which would permit the ability to intraoperatively burr down the component and “tailor” its size in the event of substantial medial overhang. Other options would have included the use of custom cutting blocks and implants; however, this would have required preoperative advanced imaging and delayed surgery because of the period of fabrication.

The patient elected to undergo her left TKA first, as it was more symptomatic. A standard anterior midline incision was used, and a medial parapatellar arthrotomy was performed. Full-thickness chondrolysis in the medial compartment was observed. A limited medial release was then performed off the proximal tibia. The Stryker Hinge Knee System instrumentation (Mahwah, NJ) was used for this procedure. The distal femoral cut was made with a 6° valgus-cut angle off an intramedullary guide. The rest of the femoral preparation was performed unconventionally given the extremely small size of the patient’s distal femur, particularly in the medial to lateral dimension. The extra small cutting block, which was initially selected, was still too large to be properly secured to the distal femur. As such, the block was held in position provisionally by hand, whereas the appropriate cuts were scored and then completed “free-hand” without a block.

**Figure 2:**
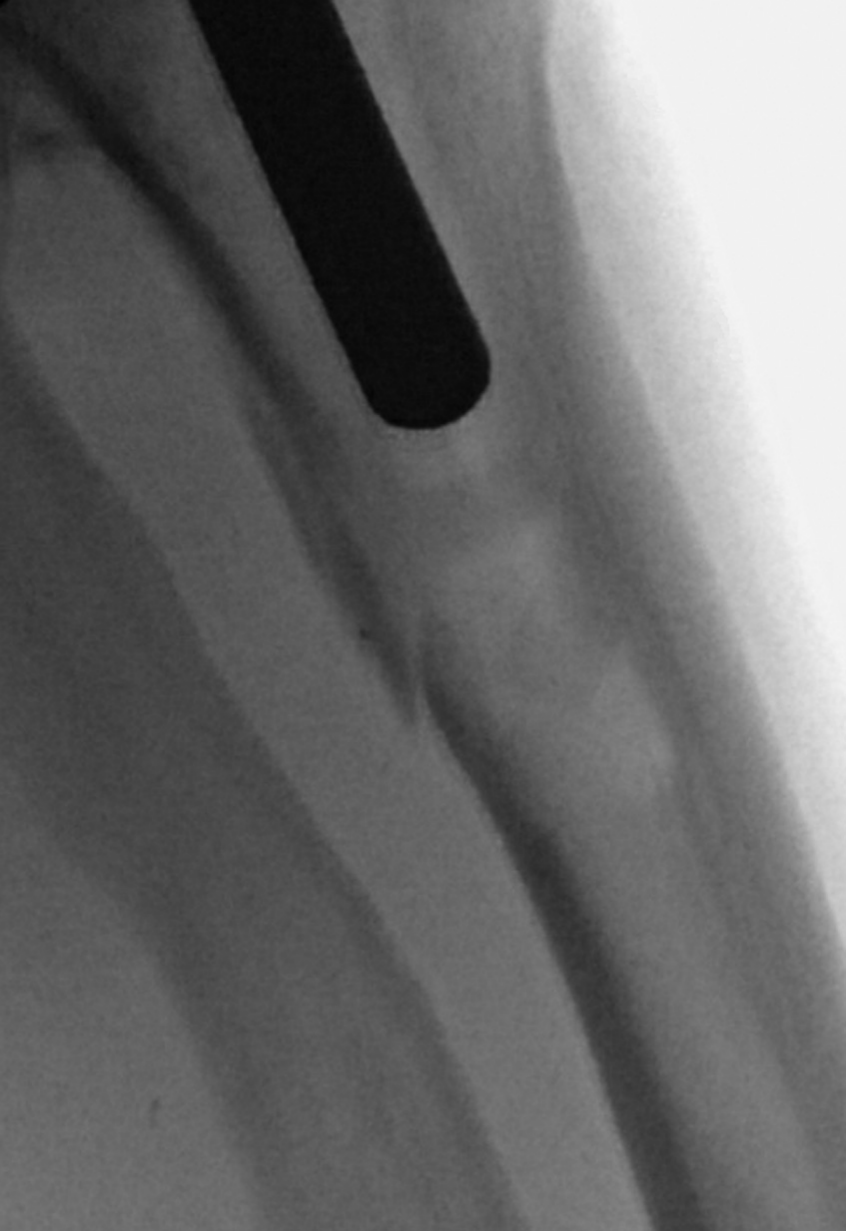
Lateral intraoperative fluoroscopy image demonstrating posterolateral metaphyseal tibia fracture that occurred during the performance of her left total knee arthroplasty.

Owing to the length of the patient’s leg, the intramedullary cutting guide was selected over the standard extramedullary cutting guide for the tibial resection. After sizing the tibial base plate, there was some resistance with the tibial punch, and the extra-small tibial trial component was noted to sit a few millimeters proud. As the knee did not come into full extension, radiographs were obtained, and they showed a nondisplaced fracture of the posterolateral tibial metaphysis (Fig. 2). The tibia was subsequently downsized to an 8-mm all-polyethylene pediatric component, which allowed for full knee extension. During trialing, the patella was noted to track centrally over the trochlea and was devoid of chondrolysis. The decision was therefore made to not resurface the patella, but rather perform a denervation procedure with associated limited lateral facetectomy.

During cementing of the components, cement restrictors and a cement gun were used. The femoral and tibial components were cemented separately in 2 cement mixes, with the tibial component being cemented before the femoral component. The hinge was assembled, and the wound closed in a standard fashion. Weight-bearing was restricted to 20 lbs foot-flat with a walker for the initial 6 weeks given her intraoperative fracture. She underwent a right TKA 3 months later having fully recovered from her left side surgery. This was performed in the same manner as the left side. To avoid intraoperative fracture of the tibia, the pediatric size tibial component was selected as the first trial. Postoperatively, she was allowed to weight bear as tolerated immediately.

She progressed well postoperatively without any complications. By her 3-month postoperative appointment after the right side, she no longer had significant pain, did not use a gait aide, and returned to work in her legal office. She was most recently seen at her 2-year postoperative appointment and continues to do well with minimal pain and no gait aides. Her only reported functional difficulty is descending stairs, for which she uses a railing. She also notes occasional nonpainful crepitus around her patellae. Upon examining, her gait was nonantalgic with resolution of the varus thrust and bilateral neutral limb alignment on standing. Her incisions were well healed, knee range of motion was 0°-95° bilaterally, and excellent stability was noted in the coronal plane throughout her entire arc of motion.

She completed patient report outcome measures at her 2-year postoperative visit. She was able to participate in moderate activity and could do unlimited housework (UCLA Activity Score 5, improved from 2 preoperatively [restricted to minimum activities of daily living]). Knee Injury and Osteoarthritis Outcome Score for Joint Replacement scores for both knees improved to 76.332 from 42.281 preoperatively. Her Oxford Knee scores improved to 42 on the left and 41 on the right compared with 9 preoperatively for both knees. Her postoperative Forgotten Joint Score for both knees was 60.4 out 100, slightly below the median of 68.8 for patients 1 year out from TKA, with normal skeletal stature [12]. Her postoperative Patient-Reported Outcomes Measurement Information System 10 scores of global physical health and mental health scores were 75% and 85%, respectively. The patient stated that she was extremely happy she had the procedures and that they greatly improved her quality of life.

**Figure 3:**
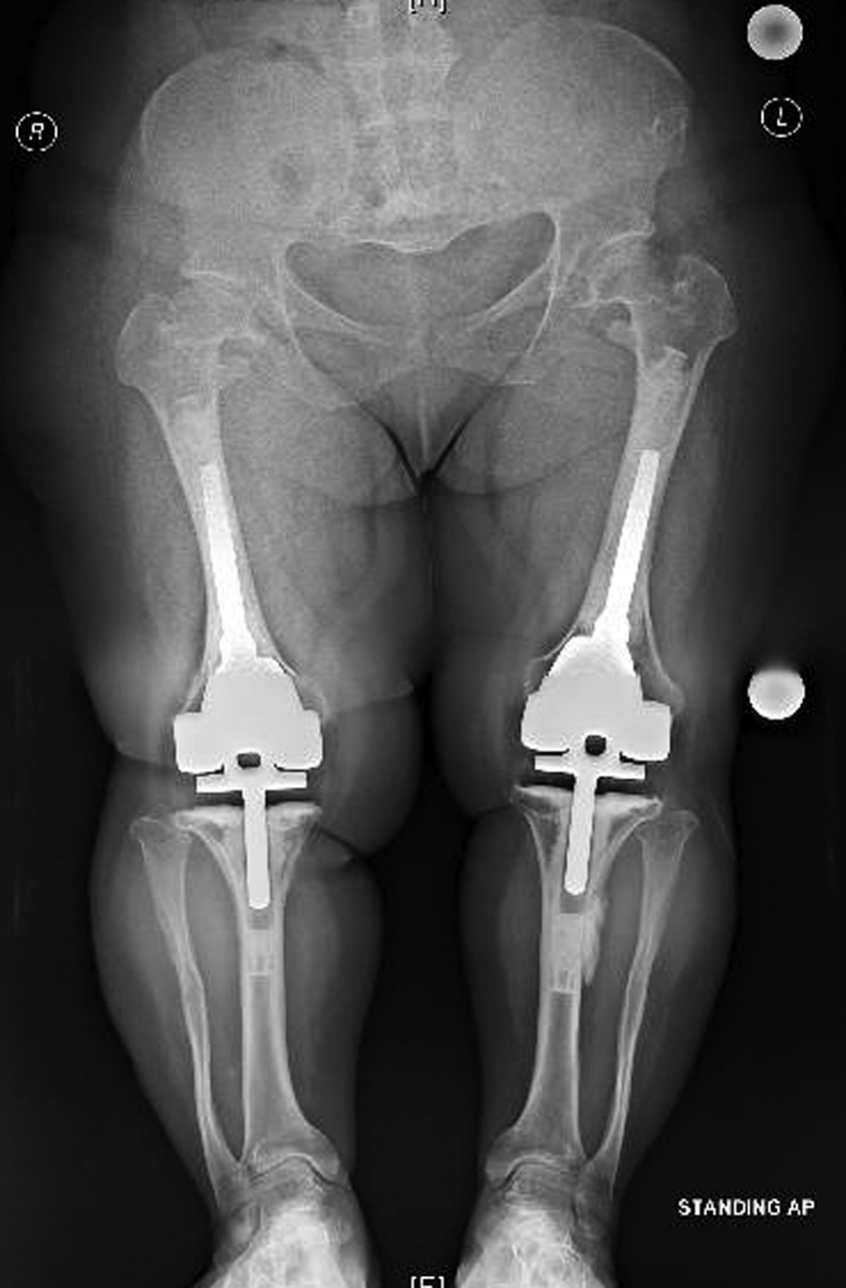
AP bone length, right and left lateral, and sunrise radiographs 2 years postoperatively with no evidence of early polyethylene wear, aseptic loosening, or stem subsidence.

**Figure 4:**
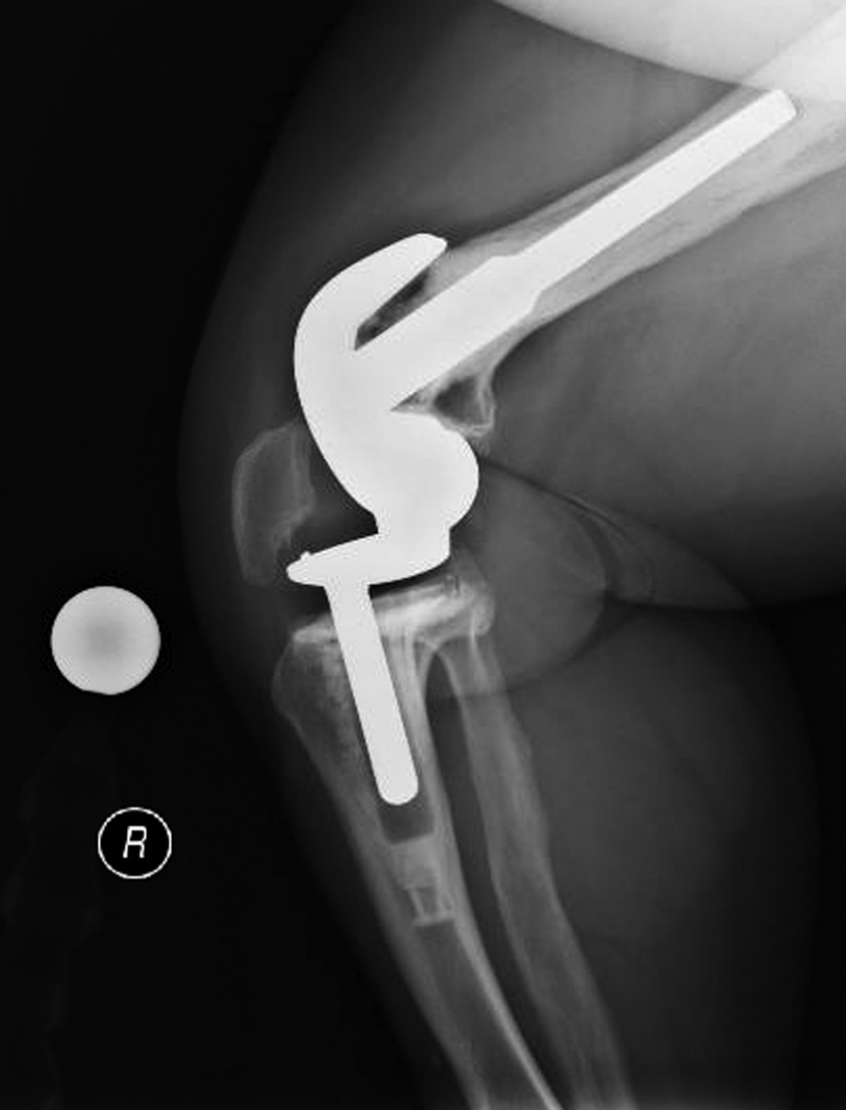
AP bone length, right and left lateral, and sunrise radiographs 2 years postoperatively with no evidence of early polyethylene wear, aseptic loosening, or stem subsidence.

**Figure 5:**
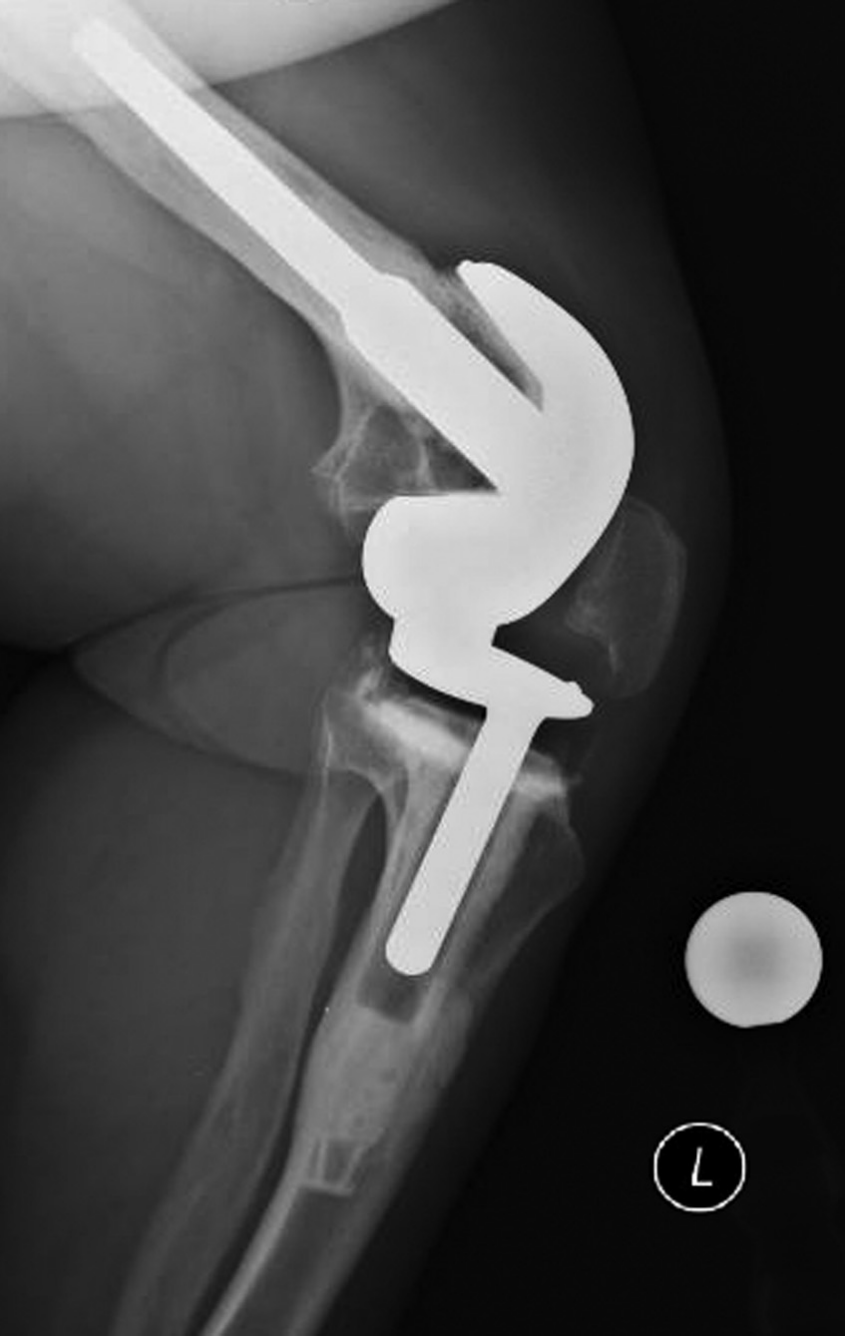
AP bone length, right and left lateral, and sunrise radiographs 2 years postoperatively with no evidence of early polyethylene wear, aseptic loosening, or stem subsidence.

**Figure 6:**
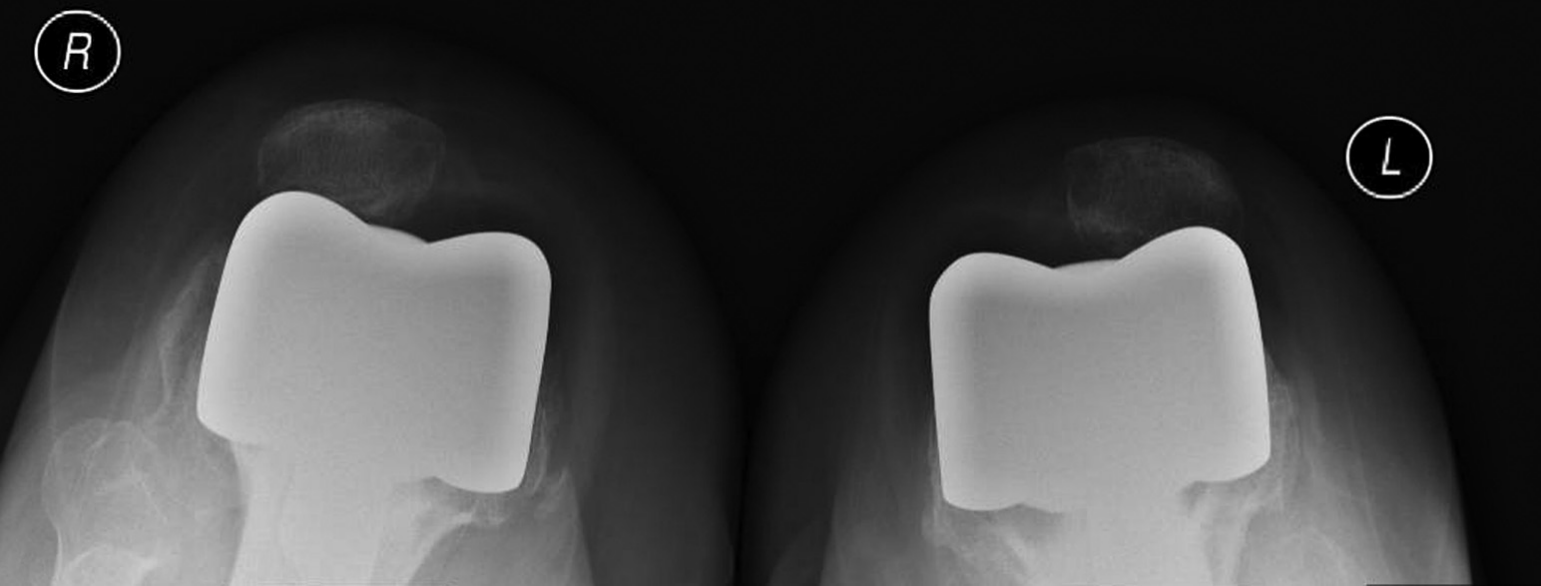
AP bone length, right and left lateral, and sunrise radiographs 2 years postoperatively with no evidence of early polyethylene wear, aseptic loosening, or stem subsidence.

The 2-year postoperative standing alignment radiographs showed maintained neutral mechanical limb axes without evidence of early polyethylene wear, aseptic loosening, or stem subsidence. Her patellar alignment and radiographically apparent patellar wear were unchanged from her 3-month postoperative radiographs (Figure 3, Figure 4, Figure 5, Figure 6). Her next follow-up visit will be at 5 years postoperatively or sooner should issues arise.

## Discussion

We report the rare presentation of a patient with achondroplasia and severe bilateral knee osteoarthritis with associated ligamentous instability. Given her failure of nonsurgical management and severe pain and disability, TKA was indicated to restore function and improve pain.

In addition to careful preoperative planning, thorough patient counseling is required when performing total joint arthroplasty in patients with skeletal dysplasias. There are increased risks of intraoperative and postoperative complications secondary to her smaller skeletal stature, metaphyseal deformity, and ligamentous laxity. Kim et al. reported 2 transient peroneal nerve palsies in their series of 12 patients who underwent TKA with a history of skeletal dysplasia [11]. Patel et al. showed that patients with skeletal dysplasia undergoing TKA were generally younger with fewer medical comorbidities than the general TKA population, although they reported higher rates of surgical site infection and acute blood loss anemia requiring transfusion [13]. Guenther et al. showed worse survival rates of TKA in patients with skeletal dysplasia than those of TKA in the general population at 5 years [14].

Technical considerations include the use of revision-style implants to allow for increased constraint and diaphyseal fixation to ensure coronal plane stability and minimize the risk for aseptic loosening. Care must be taken during templating to ensure that appropriate smaller implant sizes, including pediatric sizes, are available. Multiple options for both intramedullary and extramedullary guides should also be available during femoral and tibial preparation. Computer navigation and custom cutting blocks are also options that can be used if patient anatomy does not allow for traditional techniques such as intramedullary femoral instrumentation [11].

Patient-specific instrumentation was considered for this case. Preoperative advanced imaging allows for fabrication of custom cutting blocks that fit to the patient’s preoperative bony morphology and creates custom-sized implants. This technique also has the potential for more accurate component sizing, given the limited options available for patients with achondroplasia. There are limited data to date on using this technique in patients with achondroplasia [11]. Previous reports have demonstrated accuracy with this technique in restoring limb alignment in 10 patients with extra-articular deformities <20° not associated with skeletal dysplasia [15]. A larger study using postoperative CT scans in 1257 patients with knee osteoarthritis without significant deformity was shown to have better mechanical axis and rotational positioning in TKAs that used patient-specific instrumentation compared with conventional instrumentation [16].

These results, however, have not been found consistently in other studies and do not correlate with improved patient outcomes [17,18]. A systematic review of available literature did not demonstrate differences in overall alignment, improvement in pain, function, and overall satisfaction [19]. It additionally showed that surgeons often had to make intraoperative changes, deviating from the manufacturers’ preoperative plan [20]. With this caveat in mind, patient-specific instrumentation may have allowed for cutting blocks that would have better fit the patients' anatomy and prevented the need to free-hand a portion of our femoral preparation. However, if the custom cutting blocks or implants had not fit properly, backup standard instrumentation and implants would have still been required significantly increasing the costs and time of the case. Ultimately, the reported technique of using the traditional blocks to score the cuts and completing them with a free-hand technique worked well for this patient and did not incur any additional imaging or cost. The intraoperative left tibia fracture was caused by improper selection of initial component size. The keel on the extra small implant was too large for this patient’s anatomy. The pediatric sized implant fits appropriately.

Accelerometer-based computer navigation was also considered in this case as it has similarly been shown to be an effective tool in complex primary TKA with extra-articular deformity [21]. We attempted to use the KneeAlign2 system (Orthalign, Inc., Aliso Viejo, CA), which has been shown to reliably target neutral coronal alignment, tibial slope, and rotation within 2° in 95.7% of cases compared with 68.2% using conventional instrumentation [22,23]. In this case, however, we were unable to use the instrumentation as the patient’s tibia was too short to accommodate the malleolar referencing tibial guide. Future applications of this device in patients with skeletal dysplasias should be considered.

## Summary

Knee osteoarthritis in patients with achondroplasia is rare despite these patients commonly developing significant genu varum. In those that develop end-stage osteoarthritis, TKA is technically feasible and can significantly improve pain and function. Appropriate patient and implant selection as well as careful surgical technique to avoid intraoperative complications is paramount.

## Conflict of Interests:

The authors declare there are no conflicts of interest.

## Supplementary data

Conflict of Interest Statement for Goldberg

Conflict of Interest Statement for Stancil

Conflict of Interest Statement for Sassoon

Conflict of Interest Statement for Bouchard
