# Supplementary material for: Complex Primary Total Knee Arthroplasty in a Patient with Achondroplasia, Osteoarthritis, and Severe Coronal Instability
Source: Arthroplast Today. 2021 Feb 24;8:24–8. doi: 10.1016/j.artd.2020.12.023 (PMC7917396; doi:10.1016/j.artd.2020.12.023)
Supplement: Conflict of Interest Statement for Goldberg [file mmc1.docx]

# CONFLICT OF INTEREST STATEMENT

***American Association of Hip and Knee Surgeons***

(Adopted from the American Academy of Orthopaedic Surgeons disclosure statement)

The following form **must be filled out completely and submitted by each author (example, 6 authors, 6 forms).**

**All items require a response. If there is no relevant disclosure for a given item, enter "*None*.”**

Complex Primary Total Knee Arthroplasty in a Patient with Achondroplasia, Osteoarthritis, and Severe Coronal Instability

1. Royalties from a company or supplier (The following conflicts were disclosed) None

2. Speakers bureau/paid presentations for a company or supplier (The following conflicts were disclosed) None

3A. Paid employee for a company or supplier (The following conflicts were disclosed) None

3B. Paid consultant for a company or supplier (The following conflicts were disclosed) Consultant for a not-for-profit organization: The Schwartz Center for Compassionate Healthcare, Boston MA.

3C. Unpaid consultants for a company or supplier (The following conflicts were disclosed) None

4. Stock or stock options in a company or supplier (The following conflicts were disclosed) None

5. Research support from a company or supplier as a Principal Investigator (The following conflicts were disclosed) None

6. Other financial or material support from a company or supplier (The following conflicts were disclosed) None

7. Royalties, financial or material support from publishers (The following conflicts were disclosed) None

8. Medical/Orthopaedic publications editorial/governing board (The following conflicts were disclosed) None

9. Board member/committee appointments for a society (The following conflicts were disclosed) POSNA Wellness Committee

**Each author must sign AND print or type his/her name, date and submit a separate form**

In addition, one BLINDED Conflict of Interest form (no author names used) should be submitted per manuscript with all author disclosures.

Michael J. Goldberg Michael J Goldberg August 25. 2020

Author Name (Print or Type) Author Signature Date
