# Supplementary figures and images for: Complex Primary Total Knee Arthroplasty in a Patient with Achondroplasia, Osteoarthritis, and Severe Coronal Instability
Source: Arthroplast Today. 2021 Feb 24;8:24–8. doi: 10.1016/j.artd.2020.12.023 (PMC7917396; doi:10.1016/j.artd.2020.12.023)

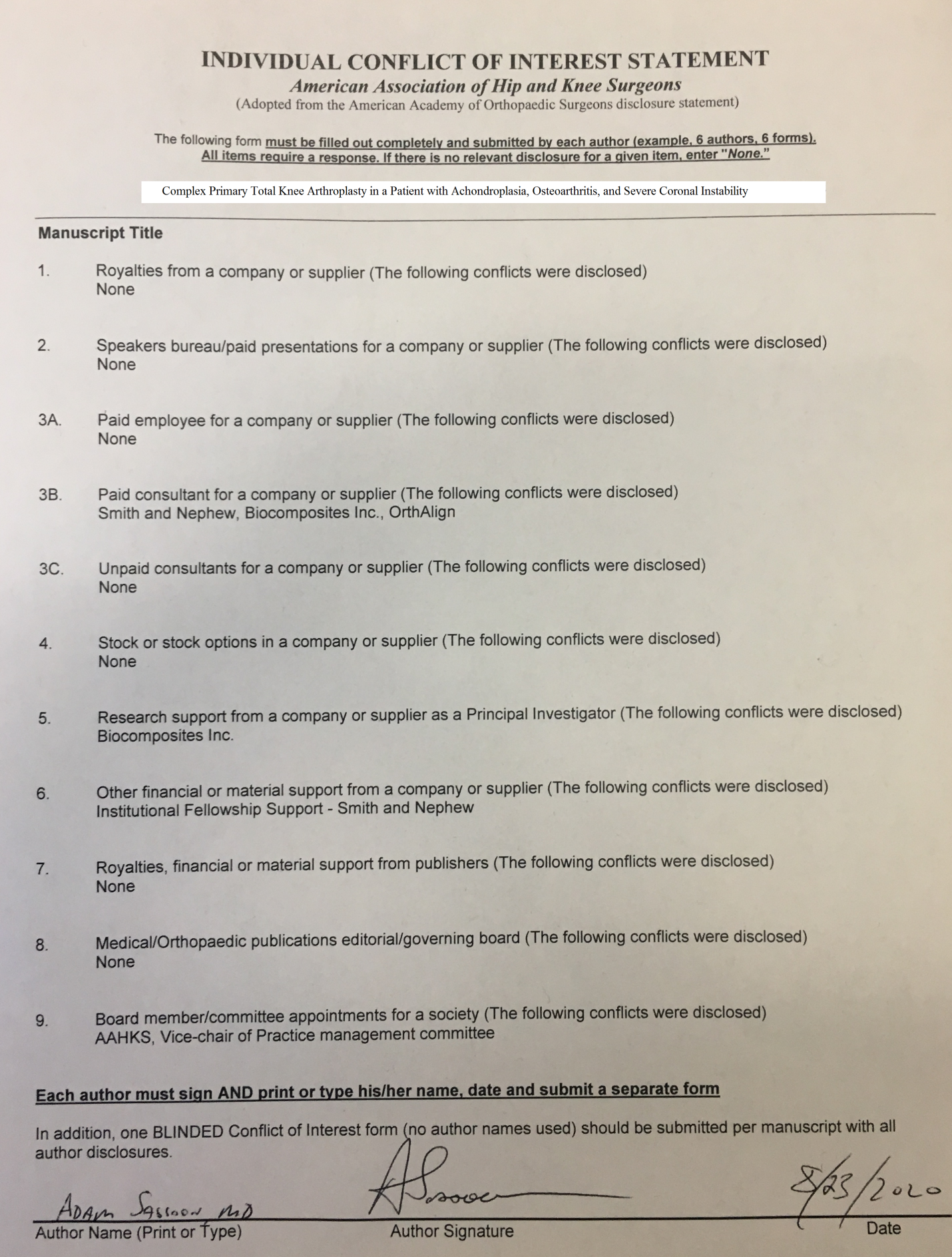

Supplement: Conflict of Interest Statement for Sassoon [file mmc3.docx]
