# Supplementary material for: Complex Primary Total Knee Arthroplasty in a Patient with Achondroplasia, Osteoarthritis, and Severe Coronal Instability
Source: Arthroplast Today. 2021 Feb 24;8:24–8. doi: 10.1016/j.artd.2020.12.023 (PMC7917396; doi:10.1016/j.artd.2020.12.023)
Supplement: Conflict of Interest Statement for Bouchard [file mmc4.docx]

# CONFLICT OF INTEREST STATEMENT

***American Association of Hip and Knee Surgeons***

(Adopted from the American Academy of Orthopaedic Surgeons disclosure statement)

The following form **must be filled out completely and submitted by each author (example, 6 authors, 6 forms).**

**All items require a response. If there is no relevant disclosure for a given item, enter "*None*.”**

Complex Primary Total Knee Arthroplasty in a Patient with Achondroplasia, Osteoarthritis, and Severe Coronal Instability

1. Royalties from a company or supplier (The following conflicts were disclosed)

2. Speakers bureau/paid presentations for a company or supplier (The following conflicts were disclosed)

3A. Paid employee for a company or supplier (The following conflicts were disclosed)

3B. Paid consultant for a company or supplier (The following conflicts were disclosed)

Orthopaedics Inc, Nuvasive Technologies, Novadip Biosciences

3C. Unpaid consultants for a company or supplier (The following conflicts were disclosed)

4. Stock or stock options in a company or supplier (The following conflicts were disclosed)

5. Research support from a company or supplier as a Principal Investigator (The following conflicts were disclosed)

6. Other financial or material support from a company or supplier (The following conflicts were disclosed)

7. Royalties, financial or material support from publishers (The following conflicts were disclosed)

8. Medical/Orthopaedic publications editorial/governing board (The following conflicts were disclosed)

9. Board member/committee appointments for a society (The following conflicts were disclosed)

**Each author must sign AND print or type his/her name, date and submit a separate form**

In addition, one BLINDED Conflict of Interest form (no author names used) should be submitted per manuscript with all author disclosures.

Maryse Bouchard
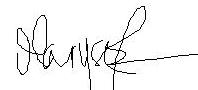
 September 1, 2020

Author Name (Print or Type) Author Signature Date
